# Predictive and Prognostic Molecular Biomarkers for Response to Neoadjuvant Chemoradiation in Rectal Cancer

**DOI:** 10.3390/ijms18030573

**Published:** 2017-03-07

**Authors:** Delphine Dayde, Ichidai Tanaka, Rekha Jain, Mei Chee Tai, Ayumu Taguchi

**Affiliations:** Departments of Translational Molecular Pathology, The University of Texas MD Anderson Cancer Center, 1515 Holcombe Blvd., Houston, TX 77030, USA; DADayde@mdanderson.org (D.D.); ITanaka@mdanderson.org (I.T.); RJain2@mdanderson.org (R.J.); MTai1@mdanderson.org (M.C.T.)

**Keywords:** rectal cancer, biomarker, neoadjuvant chemoradiation

## Abstract

The standard of care in locally advanced rectal cancer is neoadjuvant chemoradiation (nCRT) followed by radical surgery. Response to nCRT varies among patients and pathological complete response is associated with better outcome. However, there is a lack of effective methods to select rectal cancer patients who would or would not have a benefit from nCRT. The utility of clinicopathological and radiological features are limited due to lack of adequate sensitivity and specificity. Molecular biomarkers have the potential to predict response to nCRT at an early time point, but none have currently reached the clinic. Integration of diverse types of biomarkers including clinicopathological and imaging features, identification of mechanistic link to tumor biology, and rigorous validation using samples which represent disease heterogeneity, will allow to develop a sensitive and cost-effective molecular biomarker panel for precision medicine in rectal cancer. Here, we aim to review the recent advance in tissue- and blood-based molecular biomarker research and illustrate their potential in predicting nCRT response in rectal cancer.

## 1. Introduction

Colorectal cancer (CRC) is the third most commonly diagnosed cancer and the fourth leading cause of cancer-related mortality in men and women worldwide [1]. Rectal cancer accounts for approximately 30% of CRC and is associated with worse clinical outcome [2,3]. The standard treatment of locally advanced rectal cancer is neoadjuvant chemoradiation (nCRT) followed by a total mesorectal excision in order to improve resectability, anal sphincter preservation, and local control.

Clinical trials demonstrated that the local recurrence rate and toxicity were lower in rectal cancer patients with preoperative CRT compared to rectal cancer patients with postoperative CRT [4,5,6,7], while overall survival (OS) rates were not significantly different between the two groups [5,6,7]. 5-Fluorouracil (5-FU) is currently used as the standard chemotherapy agent for nCRT of locally advanced rectal cancer while additional chemotherapy agents including capecitabine and oxaliplatin recently showed promise in increasing the pathologic complete response (pCR) compared with the regimen using 5-FU [8,9]. However, the response to nCRT in locally advanced rectal cancer varies among patients. While ~40% of patients have a partial response (PR) and 8%–20% of patients achieve a pCR at the time of surgery, a subset of tumors (~20%) exhibit resistance to nCRT, demonstrating either progression or only minimal regression to stable disease [10,11,12,13,14]. These different responses to nCRT are associated with long-term outcomes including disease-free survival (DFS) and 10-year cumulative incidence of distant metastasis [15]. In addition, given the observation of pCR in a significant proportion of patients undergoing nCRT and the adverse effects of surgery (bowel, urinary and sexual dysfunctions), alternative approaches, such as the “wait-and-see” policy or transanal local excision, have been suggested [16,17,18]. On the other hand, patients exhibiting resistance to nCRT need more successful treatment approaches earlier in their management. Therefore, there is a critical need of biomarkers predicting response to nCRT at an early time point, allowing to select rectal cancer patients who would or would not have a benefit from nCRT, to reduce toxicity associated with ineffective nCRT, and to provide adequate treatment option. 

Clinical factors, including tumor size, clinical T and N stages, distance of tumor from the anal verge, and interval from nCRT to surgery, are associated with response to nCRT in rectal cancer [19,20,21,22,23]. In addition, some pathological features have been shown to predict the response to nCRT. Tumor differentiation, circumferential tumor, mucinous histology and the presence of macroscopic ulceration are associated with poor response to nCRT [20,21,22,23,24]. Imaging modalities including positron emission tomography-computed tomography, magnetic resonance imaging (MRI) and endoscopic ultrasound are currently used for pretreatment staging, assessment of response to nCRT, and restaging after nCRT [25,26,27,28,29,30,31,32]. Findings from these imaging modalities, including tumor regression rates and circumferential resection margin, can potentially predict response to nCRT in rectal cancer [30,31,32,33]. However the utility of these clinicopathological and radiological features are currently limited due to low sensitivity and specificity. 

Molecular biomarkers, either tissue- or blood-based, have the potential to predict response to nCRT at an early time point with sufficient sensitivity and specificity, although to date none have yet reached the clinic. In this review, we summarize recent advances in tissue- and blood-based molecular biomarker research for predicting nCRT response in rectal cancer. We included studies investigating association of biomarkers with not only nCRT response but also long-term outcomes such as OS and DFS. Regarding evaluation of nCRT response, several tumor regression grading (TRG) systems, including Dworak, Mandard, Ryan, Dworak/Rodel, American Joint Commission on Cancer (AJCC), and Memorial Sloan Kettering Cancer Center (MSKCC), are currently used [34,35]. For consistency, we grouped TRG grades corresponding to complete and nearly complete regression as responders (e.g., Dworak 3/4, Mandard 1/2, Ryan 1, and AJCC 0/1), and TRG grades corresponding to moderate, minimal, and no regression as non-responders (e.g., Dworak 0–2, Mandard 3–5, Ryan 2/3, and AJCC 2/3) [35].

## 2. Molecular Biomarkers in Tumor Tissues

### 2.1. DNA Mutation and DNA Methylation

Copy number alterations and mutations in *TP53* and Kirsten rat sarcoma viral oncogene homolog (*KRAS*) genes have been associated with pathological response. A meta-analysis suggested association of wild type p53 with good response to nCRT in 1830 rectal cancer patients from 30 studies [36]. Sakai et al. examined *TP53* mutation using targeted sequencing approach before and after nCRT in 20 rectal cancer patients, including 10 responders and 10 non-responders. Occurrence of *TP53* mutation after nCRT and increased p53 protein expression were observed in six out of nine non-responders [37]. *KRAS* mutations in codon 12, 13 and 16 have been also associated with response to nCRT, although the findings are controversial [38,39,40,41,42,43,44,45]. 

As a distinct molecular subtype of CRC is characterized by DNA hypermethylation in CpG-rich promoters (CpG island methylator phenotype; CIMP) [46], several studies investigated relationship of DNA methylation with response to nCRT and prognosis in rectal cancer, which were recently reviewed by Williamson et al. [47]. While most of studies examined DNA methylation in only a limited number of genes, Gaedcke et al. profiled whole genome methylation in 11 rectal cancer patients prior to nCRT with using CpG island array analyses, and 20 differentially methylated regions were validated in a sample set consisting of 61 rectal cancer patients. Further validation in two independent sample sets, consisting of 71 and 42 rectal cancer patients, was performed using MassARRAY technology for selected 10 differentially methylated regions. Although relationship of DNA methylation and response to nCRT was not investigated in the study, DNA methylation status of these regions was significantly associated with DFS in all three sample sets [48]. 

### 2.2. Gene Expression Profiles

Global gene expression profiling of tumor tissues has potential to identify gene signatures associated with response to nCRT. Watanabe et al. performed gene expression analyses using cDNA microarray on pretreatment biopsies from 52 rectal cancer patients. A 33-gene expression signature was established in the training set, consisting of 7 responders and 28 non-responders, and was validated in an independent test set, consisting of 6 responders and 11 non-responders, resulting in the predictive accuracy of 88.6% and 82.4% for training and test samples, respectively [49]. Agostini et al. examined gene expression profiles of pre-treatment biopsies from 42 rectal cancer patients consisting of 19 responders and 23 non-responders. A set of 19 genes was significantly differently expressed between responders and non-responders. The resulting logistic regression model consisting of zinc Finger Protein 160 (*ZNF160*), X-ray repair cross-complementing protein 3 (*XRCC3*), ATP dependent DNA helicase homolog (*HFM1*) and additional sex combs-like protein 2 (*ASXL2*), successfully discriminated responders and non-responders with accuracy of 95%. Knockdown of *XRCC3* by small interfering RNA (siRNA) restored sensitivity to 5-FU in HCT116 p53^−/−^ colon cancer cells, suggesting functional relevance of *XRCC3* in chemoresistance [50]. Through systems-based approach, the same group also identified seven genes (aldo-keto reductase family 1 member C3 (*AKR1C3*), C-X-C motif chemokine ligand 9 (*CXCL9*), *CXCL10*, *CXCL11*, indoleamine 2,3-dioxygenase 1 (*IDO1*), matrix metalloproteinase-12 (*MMP12*), and HLA class II histocompatibility antigen, DR α chain (*HLA-DRA*)) in immune system pathways, which can discriminate responders from non-responders [51]. Guo et al. performed bioinformatic analysis for gene expression data of 34 normal rectal tissue samples from three datasets and 72 rectal cancer samples from two datasets including 34 responders and 38 non-responders, and identified nCRT-response genes which were comprised of two sets of genes. Cancer related nCRT-response genes were involved in pathways including DNA replication, cell cycle, and DNA repair, while non-cancer related nCRT-response genes were involved in pathways of drug metabolism [52]. Another bioinformatic approach taken by Gim et al. using pre-therapeutic biopsy specimens from 77 rectal cancer patients resulted in development and validation of a three-class classification model predicting minimal response, moderate response, or complete response to nCRT [53]. Gantt et al. analyzed gene expression profiles of pre-treatment tumor biopsy specimens from 33 rectal cancer patients using cDNA microarrays [54]. Neuronal pentraxin II (*NPTX2*), one of the most differently expressed gene in the datasets, was recently validated using quantitative real time PCR in an independent set of tumor specimens from 40 rectal cancer patients in which decreased *NPTX2* gene expression levels was associated with improved response to nCRT and prognosis [55].

### 2.3. Proteins and Metabolites

Expression of proteins including epidermal growth factor receptor (EGFR), vascular endothelial growth factor (VEGF), p21, BCL2-associated X protein (Bax), B-cell CLL/lymphoma 2 (Bcl2), marker of proliferation Ki-67 (ki-67), p53, cyclooxygenase-2 (COX-2), hypoxia-inducible factor 1-α (HIF1-α), thymidylate synthase, E-cadherin, matrix metalloproteinase-9 (MMP-9) and matrix metalloproteinase-2 (MMP-2), have been previously associated with response to nCRT [33,56,57]. Protein biomarkers in tissues have been extensively investigated and the findings were summarized in Table 1. These newly identified protein biomarkers are involved in pathways dysregulated by chemoradiation, including DNA repair (X-ray repair cross-complementing protein 2 (XRCC2) [58], ataxia telangiectasia mutated (ATM) [59], meiotic recombination 11 homolog A (MRE11) [59], PCNA-associated factor 15 (Paf15) [60]), cell cycle (polo-like kinase 1 (Plk1) [61], and vaccinia-related kinase-1 and -2 (VRK1 and VRK2) [62]), cell proliferation (c-MYC and proliferating cell nuclear antigen (PCNA) [63], golgi phosphoprotein 3 (GOLPH3) [64], focal adhesion kinase (FAK) [65], fibroblast growth factor receptor 4 (FGFR4) [66], and nuclear factor-κB (NF-κB) [67]), apoptosis (survivin [68], and apoptotic protease-activating factor 1 (APAF-1) and COX2 [69]), autophagy (Beclin 1 [70]), cell adhesion (Plectin-1 (PLEC1) [71] and desmoglein 3 (DSG3) [72]), cell motility (transgelin (TAGLN) [71]), and metabolism (vascular non-inflammatory molecule 1 (VNN1) [73], transketolase (TKT) and hydroxyacyl-CoA dehydrogenase (HADHA) [71], and 17-β-hydroxysteroid dehydrogenase type 2 (HSD17B2) and 3-hydroxy-3-methylglutaryl coenzyme A synthase (HMGCS2) [74]). Li et al. developed a three-protein biomarker panel consisting of c-MYC, PCNA, and tissue inhibitor of metalloproteinases 1 (TIMP1) for prediction of prognosis in 329 locally advanced rectal cancer patients. Of note, combination of the biomarker panel with an imaging feature, MRI­detected extramural vascular invasion, significantly improved the prediction of OS in rectal cancer (*p* < 0.01) [63], suggesting potential of biomarkers to complement radiologic feature. In addition, functional roles of some proteins in sensitivity to chemoradiation were confirmed in vitro, allowing to mechanistically link biomarker candidates and rectal cancer. Knockdown of XRCC2 in SW480 colorectal cells enhances the sensitivity to radiation [58]. Silencing of FGFR4 using siRNA resulted in decreased survival in a DNA repair protein RAD51 homolog 1 (RAD51)-mediated manner in radioresistant HT29 cells [66]. Constitutive NF-κB activity was associated with resistance to radiation in three CRC cell lines [75].

Few studies have investigated potential of metabolites in predicting response to nCRT or prognosis in rectal cancer. Redalen et al. performed metabolite profiling using high-resolution magic angle spinning magnetic resonance spectroscopy (HR MAS MRS) on biopsies samples from 54 locally advanced rectal cancer patients who underwent nCRT followed by surgery. No significant correlation was observed between ten quantified metabolites and the Tumor Node Metastasis (TNM) classification, disseminated tumor cells, clinical stages or tumor regression grade. However, high concentration of glycine, creatinine and myo-inositol in tumors were significantly associated with progression free survival in the univariate Cox regression analysis. Multivariate regression analysis revealed that high concentration of glycine was the most significant variable associated with poor progression free survival (hazard ratio = 4.4, 95% CI = 1.4–14.3, *p* = 0.008), suggesting glycine as the prognostic biomarker in rectal cancer [76].

### 2.4. Tumor Immune Microenvironment

While activation of host immune response plays an important role in the therapeutic effects of chemoradiation, it can also activate immune suppressive pathways [77]. To determine predictive and prognostic effects of tumor immune microenvironment, Teng et al. investigated the subset densities of tumor-infiltrating lymphocytes (TILs), as well as programmed cell death ligand 1 (PD-L1) and cytotoxic T-lymphocyte protein 4 (CTLA4) expression before and after nCRT in 62 rectal cancer. Patients with high CD8+ TILs, high CD4+ TILs, and low Myeloid-Derived Suppressor Cells (MDSCs) achieved good response to nCRT (*p* = 0.022, 0.022 and 0.005, respectively) [78]. McCoy et al. evaluated the relationship of subset densities of TILs in post-nCRT surgical samples from 128 rectal cancer patients. Low stromal Foxp3+ cell density was significantly associated with pCR (adjusted odds ratio = 5.27, *p* = 0.006) and improved recurrence-free survival (hazard ratio = 0.46, *p* = 0.03) [79]. 

Relationship of PD-L1 expression with response to nCRT is not consistent among studies, possibly due to some technical and biological issues, including inappropriate tumor sampling, variable PD-L1 primary antibodies, different thresholds for PD-L1 positivity, variability of tissue preparation and processing, dynamic and heterogeneous PD-L1 expression, and PD-L1 expression in tumor cells versus infiltrating immune cells [80,81]. PD-L1 was rarely detected in the study of Teng et al [78]. Hecht et al. examined PD-L1 expression in 103 pre-nCRT biopsies and 159 post-nCRT surgical specimens including 63 matched samples, and suggested that PD-L1 was upregulated after nCRT, and that low PD-L1 expression on both tumor and inflammatory cells, either at pre-nRCT (hazard ratio = 0.438, *p* = 0.045) or post-nRCT (hazard ratio = 0.257, *p* = 0.030), was an independent negative prognostic marker [82]. On the other hand, immunohistochemistry study using tumor specimens from 90 rectal cancer patients after nCRT suggested that patients with high PD-L1 expression after nCRT more frequently had vascular invasion and tumor recurrence compared to patients with low PD-L1 expression, and high PD-L1 expression was significantly associated with poor recurrence-free and overall survival (*p* = 0.003 and *p* = 0.036, respectively) [83]. 

### 2.5. MicroRNA

As previously reviewed [33,56,84,85,86], miRNAs, including miR-1183, miR-1224-5p, miR-1246, miR-125a-3p, miR-125b, miR-125-b1, miR-1274b, miR-1290-3p, miR-145, miR-1471, miR-153, miR-16, miR-188-5p, miR-190b, miR-1909, miR-196b, miR-200c, miR-205-5p, miR-21, miR-21-5p, miR-215, miR-29b-2, miR-450a, miR-450b-5p, miR-483-5p, miR-490, miR-519c-3p, miR-561, miR-590-5p, miR-622, miR-630, miR-671-5p, miR-720, miR-765, miR-99, miR-99a, let-7c, and let-7e, have been associated with response to nCRT. Gaedcke et al. examined microRNA profiles of tumor biopsies and normal mucosa from 57 rectal cancer patients before treatment using LNA-enhanced miRCURY microarray, resulting in identification of 49 differentially expressed miRNA between normal and cancer. Interestingly a set of microRNAs (miR-492, miR-542-5p, miR-584, miR-483-5p, miR-144, miR-2110, miR-652, miR-375, miR-147b, miR-148a, miR-190, miR-26a/b, and miR-338-3p) showed a distinctly different expression pattern from colon cancer. In addition, expression levels of miR-135b were significantly correlated with tumor regression grade (correlation = 0.2; 95% CI, 0.04 to 0.36; *p* = 0.01) and disease-free survival and cancer-specific survival (*p* = 0.042 and *p* < 0.001, respectively) [87]. Carames et al. examined miR-21 expression by quantitative real-time RT-PCR in biopsy samples from 76 rectal cancer patients. miR-21 was overexpressed in 54 patients and a receiver operating characteristic ROC curve analysis revealed that high miR-21 expression yielded an area under the curve (AUC) of 0.78 (95% CI = 0.56–0.99), with sensitivity of 86.6% and specificity of 60% in distinguishing pCR from non-pCR [88]. 

## 3. Biomarkers in Blood

### 3.1. Protein and Metabolites

As carcinoembryonic antigen (CEA) is a broadly recognized biomarker for prognosis and disease monitoring in CRC, relationship of CEA and response to nCRT has been well studied (Table 2) [33,56,57,85]. Although more than half of the studies used 5 ng/mL as the cutoff values of CEA (Table 2), there has been no validated cutoff values for an elevated CEA levels with sufficient sensitivity and specificity. Probst et al. recently investigated association of pre-nCRT CEA levels with response to nCRT as well as overall survival in 18,113 locally advanced rectal cancer patients from a total of 136,840 rectal cancer patients who were present in the 2006–2011 National Cancer Data Base. Forty-seven percent of the patients had elevated CEA levels before nCRT, and elevated pre-nCRT CEA was independently associated with decreased pCR (odds ratio = 0.65, 95% CI = 0.52–0.77, *p* < 0.001), reduced pathological tumor regression (odds ratio = 0.74, 95% CI = 0.67–0.70, *p* < 0.001), reduced tumor downstaging (odds ratio = 0.77, 95% CI = 0.63–0.92, *p* < 0.001), and overall survival (hazard ratio = 1.45, 95% CI = 1.34–1.58, *p* < 0.001). The positive predictive value and negative predictive value of an elevated pre-nCRT CEA for incomplete pathological response was 91% and 17%, respectively [89]. Consistent with the findings, other recent studies have also shown that pre-nCRT CEA levels were significantly associated with pCR and survival [21,90,91], while low CEA levels after nCRT was suggested as an independent predictor of pCR. 

Zhang et al. evaluated pre-treatment serum level of carbohydrate antigen 19-9 (CA19-9) and CEA in 303 locally advanced rectal cancer patients with nCRT. While serum CEA levels were not significantly different in this study, elevated serum CA19-9 levels were significantly correlated with poor OS (*p* = 0.003), DFS (*p* = 0.001), and distant metastasis-free survival (*p* = 0.039). Patients with both higher CA19-9 and CEA levels had the worst OS (*p* = 0.021) and DFS (*p* = 0.006) [110].

Fibrinogen is one of acute-phase proteins and often elevated in various types of cancer patients. Lee et al. examined levels of fibrinogen in plasmas from 947 rectal patients who received nCRT [111]. Elevated fibrinogen levels as well as CEA levels before nCRT was a significant predictive factors for downstaging, primary tumor regression, and pCR. The model combining CEA (cut off values of 5.0 ng/mL) and fibrinogen (cut off values of 270 mg/dL) discriminated pCR from non-pCR with sensitivity of 34.6% and specificity of 83.3%.

Hektoen et al. assessed serum carbonic anhydrase 9 (CAIX) collected from 66 rectal cancer patients at four different time points: before nCRT, after two cycles of nCRT, at nCRT completion, and four weeks after nCRT completion. Interestingly serum CAIX levels increased along with nCRT and returned toward baseline four weeks after nCRT completion. With applying threshold of 224 pg/mL for ΔnCRT (increase after two cycles of nCRT compared to baseline), sensitivity, specificity, positive predictive value, and negative predictive value for predicting progression free survival was 96%, 39%, 94%, and 44%, respectively. ΔnCRT was also significantly associated with histological downstaging, although ΔnCRT did not correlate with histologic tumor regression grade [112].

Crotti et al. profiled circulating peptides in the plasma from 33 rectal cancer patients who had nCRT through peptidomic methodology. Three peptides at *m*/*z* 1082.552, 1098.537, and 1104.538, selected by Random Forest classification, were able to correctly discriminate between responders (*n* = 16) and non-responders (*n* = 17) before nCRT, with an accuracy of 86% and an AUC of 0.92 [113].

With using MALDI-TOF MS, Kim et al. analyzed metabolites in serum samples collected before nCRT from 73 locally advanced rectal cancer patients. Two of the top-ranked low-mass ions were identified as hypoxanthine (HX) and phosphoenolpyruvic acid (PEP). High HX concentration and low PEP concentration in plasma were significantly associated with tumor regression grade. High HX concentration was also significantly associated with post-nCRT pathological stage [114].

### 3.2. MicroRNA

D’Angelo et al. performed miRNA microarrays analysis on biopsy specimen collected before nCRT from 38 rectal cancer patients. Among eleven miRNAs that were significantly different between responders and non-responders (increased in non-responders: miR-154, miR-409-3p, miR-127-3p, miR-214*, miR-299-5p and miR-125b; decreased in non-responders: miR-33a, miR-30e, miR-338-3p, miR-200a and miR-378), levels of miR-125b was further examined in sera from 34 locally advanced rectal cancer patients. Serum miR-125b levels were significantly higher in non-responders that responders (*p* = 0.009), with an AUC of 0.782 (95% CI = 0.612–0.952) in discriminating responders from non-responders [115]. 

Yu et al. profiled microRNA expression using miRNA microarray in tumor tissues from three nCRT-sensitive and three nCRT-resistant rectal cancer patients before treatment. miR-345 was significantly increased in tumor tissues from nCRT-resistant rectal cancer patients and therefore levels of miR-345 was evaluated in two independent serum sample sets consisting of 87 and 42 locally advanced rectal cancer patients, respectively. Serum miR-345 levels were significantly higher in non-responders in both sample sets (*p* = 0.002, AUC = 0.69 (95% CI: 0.57–0.80); *p* = 0.007, AUC = 0.75 (95% CI: 0.57–0.93)), and low serum miR-345 levels were associated with superior three-year local recurrence free survival (hazard ratio = 0.14, 95% CI = 0.04–0.49, *p* = 0.002) [116]. 

### 3.3. Circulating Tumor Cells (CTCs)

Accumulating evidence indicates CTCs as promising biomarkers for prediction of prognosis and relapse and disease monitoring in various types of epithelial cancers including CRC [117]. Sun et al. identified CTCs using epithelial cell-adhesion molecule (EpCAM) magnetic bead-based enrichment combined with cytometric approach in all 103 rectal cancer patients, while no CTCs were identified in 25 healthy controls. For 50 locally advanced rectal cancer patients, the levels of CTCs were significantly decreased after nCRT (5.82 ± 3.37 for pre-nCRT, 1.98 ± 2.50 for post-nCRT; *p* < 0.001). Interestingly, significant difference was observed in the levels of post-CRT CTCs and ∆%CTC (percentage difference in CTC levels between pre-CRT and post-CRT) between responders and non-responders, with AUC of 0.771 for post-CRT CTC levels and 0.806 for ∆%CTC [118]. Sun et al. also identified and counted cytokeratin+/CD45−/DAPI+ CTCs in 115 locally advanced rectal cancer patients with using size-based microfluidic device [119]. While the baseline CTC counts of 64 responders were significantly higher than those of 51 non-responders (44.50 ± 11.94 vs. 37.67 ± 15.45, *p* = 0.012); responders had significantly lower CTC counts after nCRT than non-responders (3.61 ± 2.90 vs. 12.08 ± 7.40, *p* < 0.001). ROC curve analysis yielded AUC of 0.860 for ∆%CTC in discriminating responders from non-responders [120]. CEA was not significantly associated with response to nCRT in these two studies. Magni et al. identified CTCs using the CellSearch System in peripheral blood collected before nCRT from 16 (18.8%) of 85 rectal cancer patients. CTCs were decreased in blood samples collected after nCRT from responders while no significant change was observed in non-responders [121]. Two studies assessed mRNA expression of cytokeratin-20 (*CK20)* in blood mononuclear cells [122] and *CEA/CK20/CD133* in peripheral blood [123] using RT-PCR to identify CTCs in rectal cancer. Although PCR-based CTC detection does not directly observe and enumerate CTCs and therefore might be less specific compared to immunocytologic methods [117], detection rates of these markers were significantly associated with nCRT response [122] and local recurrence [123].

### 3.4. Circulating Cell-Free Nucleic Acids 

As previously reviewed [57,85], circulating cell-free nucleic acids as well as CTCs, can be a promising material for liquid biopsy in rectal cancer. Sun et al. examined *KRAS* mutation and O6-methylguanine-DNA methyltransferase (*MGMT*) promoter methylation in plasma cell-free DNA (cfDNA) from 34 locally advanced rectal cancer patients. The concentration of baseline cfDNA in patients with rectal cancer was significantly higher compared to 10 healthy controls. While detection rate of *KRAS* mutation decreased significantly after nCRT in both responders and non-responders, *MGMT* promoter methylation in cfDNA at baseline was significantly higher in responders compared to non-responders (*p* = 0.04) [124].

### 3.5. Host Immune Response

Cytokine including interleukin (IL)-6 and IL-8 have been associated with nCRT in rectal cancer [85]. Tada et al. examined concentration of IL-2, IL-4, IL-6, IL-10, interferon-gamma (IFN-γ), tumor necrosis factor-α (TNF-α), monocyte chemoattractant protein-1 (MCP-1), C-C motif chemokine ligand-5 (CCL-5), TNF-related apoptosis-inducing ligand (TRAIL) and soluble CD40-ligand, using a bead-based multiplex immunoassay in the plasma samples collected from 35 rectal cancer patients before and after nCRT. While none of cytokine levels before nCRT were significantly associated with response to nCRT, levels of IL-6 and TNF-α after nCRT were significantly higher in non-responders compared to responders, and also significant decrease of soluble CD40-ligand and CCL-5 after nCRT was observed in responders [125].

Neopterin is a pteridine compound produced by macrophages activated by IFN-γ. Zezulova et al. found that pre-nCRT serum neopterin at the cutoff of 3 μg/L was a significant predictor of poor relapse free survival (*p* = 0.001) and OS (*p* < 0.001) in 28 rectal cancer patients treated with nCRT [126].

Several studies have evaluated the relationship of neutrophil-to-lymphocyte ratio (NLR) with response to nCRT [85]. Caputo et al. recently examined NLR and derived neutrophil-to-lymphocyte ratio (d-NLR) before and after nCRT in 87 rectal cancer patients. Higher NLR and d-NLR after nCRT was significantly associated with poor response to nCRT and postoperative complications [127]. Dong et al. conducted a meta-analysis which included seven cohorts involving 959 patients to evaluate the prognostic value of NLR in patients with rectal cancer. Their pooled results demonstrated that elevated NLR was associated with poor OS (hazard ratio = 13.41, 95% CI = 4.90–36.72), DFS (hazard ratio = 4.37, 95% CI = 2.33–8.19), and recurrence-free survival (hazard ratio = 3.64, 95% CI = 1.88–7.05) [128]. Dreyer et al. assessed the association of systemic inflammation, including Hemoglobin, CEA, NLR, d-NLR, platelet-to-lymphocyte ratio (PLR), neutrophil-platelet score (NPS), and the modified Glasgow prognostic score (mGPS), which is determined by the levels of C-Reactive protein (CRP) and albumin, with the response to nCRT in 79 rectal cancer patients. mGPS was significantly associated with a poor pathologic response to nCRT (*p* = 0.022) [129]. 

## 4. Single Nucleotide Polymorphisms (SNPs)

Previous studies, reviewed elsewhere [57,130], have indicated the potential association between response to nCRT in rectal cancer and SNPs in several genes including *TS*, *EGFR*, epidermal Growth Factor (*EGF*), coiled-coil protein DIX1 (*CCD1*), superoxide Dismutase 2 (*SOD2*), *IL13*, 8-oxoguanine DNA glycosylase (*OGG1*) and methylenetetrahydrofolate reductase (*MTHFR*). To our knowledge, only one genome-wide study has explored association of SNPs with response to nCRT in rectal cancer [131]. Genome-wide genotyping in 43 rectal cancer patients using SNP array resulted in identification of nine SNPs as nCRT-responsive SNPs and association of coronin 2A (*CORO2A*) rs1985859 with nCRT response was validated in 113 rectal cancer patients [131]. Nikas et al. indicated in 108 rectal cancer patients that homozygous C/C genotype in *MTHFR* gene (rs1801133) were 2.91 times more likely to respond to nCRT (*p* = 0.015) and 3.25 times more likely not to have recurrence (*p* = 0.008) than either the heterozygous C/T or homozygous T/T genotype [132], which is consistent with previous study [133]. Nelson et al. investigated SNP located in the promoter region of the *TS* gene, which potentially affect the enzymatic activity and catabolism of 5-FU, in 50 rectal cancer patients and found that patients with at least one thymidylate synthase 3G allele were more likely to have a complete or partial pathologic response to nCRT containing 5-FU, with odds ratio of 10.4 (95% CI = 1.3–81.6, *p* = 0.01), than those without [134]. In addition, xeroderma pigmentosum group G (*XPG*) C46T rs1047768 and mutS homolog 6 (*MSH6*) rs3136228 have been significantly associated with response to nCRT in rectal cancer [135,136].

Recent studies also suggest the potential relationship of nCRT response and SNPs in the genes associated with microRNA. The genes SMAD family member 3 (*SMAD3*), trans-activation-responsive RNA-binding protein (*TRBP*) and double-stranded RNA-specific endoribonuclease (*DROSHA*) play a crucial roles in microRNA processing [137] and SNPs in these genes (rs745103, rs744910 and rs1722821 for *SMAD3*; rs6088619 for *TRBP*; and rs10719 for *DROSHA*) were significantly associated with nCRT response in 265 rectal cancer patients [138]. The occurrence of SNPs located in miRNA target sites may affect interaction of miRNA and target genes and may modulate target gene expression, potentially affecting risk and clinical outcome of CRC. Vymetalkova et al. examined 13 SNPs in predicted miRNA target sites in mucin genes in 310 rectal cancer patients and the patients with CC genotype of rs4729655 in mucin 17 (*MUC17*) gene exhibited a longer overall survival (hazard ratio = 0.27, 95% CI = 0.14–0.54, *p* < 0.001) than those without CC genotype [139]. The same research group also identified significant association of overall survival in CRC with SNPs located in miRNA target sites of DNA repair protein RAD52 homolog (*RAD52*) (rs11226), X-ray repair cross-complementing protein 5 (*XRCC5*) (rs1051685) and single-strand-selective monofunctional uracil-DNA glycosylase 1 (*SMUG1*) (rs2233921) [140,141]. 

## 5. Conclusions

Despite nearly a decade after the establishment of nCRT in rectal cancer, we lack the ability to distinguish nCRT-resistant patients and sensitive patients prior to or early in the course of treatment to guide adaptive modifications to the treatment plan. Although a number of molecular biomarkers have been proposed as predictors of response to nCRT, none of them has reached the clinic. The current paucity of predictive biomarkers for nCRT in rectal cancer can be attributed to several issues in our approach to molecular biomarkers.

First, most of studies evaluate one type of biomarkers, whether consisting of gene expression profiles, proteins, microRNAs, or other types, without an appreciation of their performance relative to other markers and with an insufficient sensitivity and specificity by themselves. Thus, there is a need to compare and integrate different types of biomarkers from multiple biomarker platforms, including clinicopathological and imaging features, side by side in the same sample sets to determine their comparative performance and the contribution of individual biomarkers to a robust panel. Such an integrative approach will allow us to make the best choice of biomarkers to develop an optimal and reliable biomarker model that can predict response to nCRT more accurately. Examples of successful biomarker integration include tissue expression of three proteins (c-MYC, PCNA, and TIMP1) combined with MRI­detected extramural vascular invasion [63] and the modified Glasgow prognostic score (mGPS; combination of CRP and albumin) [129]. CTCs also may be promising biomarkers as the performance of CTCs in predicting nCRT response was superior to CEA in the same sample sets [118,120]. 

Second, CRC is a highly heterogeneous disease. The CRC Subtyping Consortium has proposed molecular classification of CRC, consisting of four subtypes. DNA mutation, epigenetic features, oncogenic pathway activation, and tumor immune microenvironment were distinct among subgroups, and a subgroup with mesenchymal feature carried worst overall survival [142]. In addition, several studies have demonstrated high intra-tumor heterogeneity in CRC [143,144]. Based on these findings, single biomarker unlikely achieves adequate sensitivity and specificity in predicting nCRT response in rectal cancer patients with different molecular features, and therefore it is particularly important to understand the mechanistic links between biomarkers and tumor biology, adding substantial information of biomarkers and their potential limitation and allowing us to select molecular biomarkers most associated with particular disease subtypes. Patient-derived cancer cell lines serve as excellent tools for understanding mechanistic insights of biomarkers. In vitro experiments using cell lines revealed potential functions of some biomarkers, including XRCC3 [50], XRCC2 [58], FGFR4 [66], and NF-κB [75], in chemoradiation sensitivity. Interestingly, two nCRT-responsive SNPs located in miRNA target sites of the *MRE11A* gene (rs2155209) [140] and the *SMUG1* gene (rs2233921) [141] affected gene transcription. Although miRNAs that target these SNPs were not identified, the results suggested potential biological links of these SNPs to nCRT response. While cell lines derived from colon cancer patients are used in vast majority of studies, there are distinct clinical and molecular differences between colon and rectal cancer [145,146]. Given that cancer cell lines recapitulate the genetic and epigenetic background of the individual tumors, cell lines derived from rectal cancer might be more appropriate tools for biological research in rectal cancer. In order to mechanistically tie the occurrence of blood-based biomarkers to tumor biology, it is critical to understand whether biomarkers would emanate from tumors. In addition, tumors by themselves represent important materials for blood-based biomarker discovery. For example, increased miR-125b and miR-345 levels in tumors have been associated with non-responders and levels of miR-125b and miR-345 in serum were significantly higher in non-responders [115,116]. 

Third, there are significant variabilities in treatment schedules including chemotherapy regimens, radiation doses, and interval between nCRT and surgery, which may lead to different response to nCRT. Using different TRG systems may also be a potential bias among studies. Indeed, various TRG systems are used among the studies referred in this review. While the five-tier TRG systems including the Mandard and the Dworak are used in 68% of the studies, the four-tier and three-tier TRG systems are used in 20% and 12%, respectively. Among the several TRG systems currently used, Trakarnsanga et al. indicated that the four-tier AJCC system most accurately predicted recurrence in 563 rectal cancer patients treated with standard nCRT followed by surgery [34]. These clinical variabilities may increase number of samples required for statistical analyses and impede to draw clear conclusion. Therefore future studies should be rigorously designed to have adequate number of samples, following the same nCRT regimens and standardized tumor response evaluation system.

In conclusion, although no predictive molecular biomarkers for response to nCRT is sufficiently robust to have clinical utility, integration of diverse types of biomarkers including clinicopathological and imaging features, identification of mechanistic links to tumor biology, and rigorous validation using samples which represent disease heterogeneity, will allow to develop a sensitive and cost-effective molecular biomarker panel and enhance efforts for personalized care in rectal cancer.

## Figures and Tables

**Table 1:** Tissue-based protein biomarkers for nCRT response in rectal cancer.

Author (Reference)	Year	Number of Samples	Specimens Collection	Biomarker Name	*p*-Value
Croner et al. [71]	2016	20	pre-nCRT	PLEC1, HADHA, TKT and TAGLN	Not Reported
Qin et al. [58]	2015	67	pre-nCRT	XRCC2	*p* < 0.001
Voboril et al. [67]	2016	50	pre-nCRT and post-surgery	NF-κB	Not Significant
Lee et al. [74]	2015	172	pre-nCRT	HSD17B2 and HMGCS2	*p* < 0.001
Chai et al. [73]	2016	172	pre-nCRT	VNN1	*p* = 0.001
Chao et al. [72]	2016	46	pre-nCRT	DSG3	*p* = 0.001
Ho et al. [59]	2016	54	post-surgery	ATM and MRE11	*p* = 0.011
Cebrian et al. [61]	2016	75	pre-nCRT	PLK1	*p* = 0.049
Zhu et al. [64]	2016	148	pre-nCRT	GOLPH3	*p* = 0.026
Yan et al. [60]	2016	105	pre-nCRT	PAF15	Not Reported
Zaanan et al. [70]	2015	96	pre-nCRT	Beclin 1	*p* = 0.02
del Puerto-Nevado et al. [62]	2016	67	pre-nCRT	VRK1 and VRK2	*p* = 0.004
Gomez del Pulgar et al. [65]	2016	73	pre-nCRT	FAK	*p* = 0.007
Ahmed et al. [66]	2016	43	pre-nCRT	FGFR4	*p* = 0.03
Peng et al. [69]	2016	82	pre-nCRT and post-surgery	APAF-1 and COX-2	*p* = 0.05
Li et al. [63]	2016	329	pre-nCRT	c-Myc, PCNA and TIMP1	Not Reported
Yu et al. [68]	2016	116	post-surgery	Survivin	Not Reported

Abbreviation: nCRT, neoadjuvant chemoradiation; PLEC1, Plectin-1; HADHA, hydroxyacyl-CoA dehydrogenase; TKT, transketolase; TAGLN, transgelin; XRCC2, X-ray repair cross-complementing protein 2; NF-κB, nuclear factor-κB; HSD17B2, 17-β-hydroxysteroid dehydrogenase type 2; HMGCS2, 3-hydroxy-3-methylglutaryl coenzyme A synthase; VNN, vascular non-inflammatory molecule 1; DSG3, desmoglein 3; ATM, ataxia telangiectasia mutated; MRE11, meiotic recombination 11 homolog A; Plk1, polo-like kinase 1; GOLPH3, golgi phosphoprotein 3; Paf15, PCNA-associated factor 1; VRK1 and 2, vaccinia-related kinase 1 and 2; FAK, focal adhesion kinase; FGFR4, fibroblast growth factor receptor 4; APAF-1, apoptotic protease-activating factor 1; COX-2, cyclooxygenase-2; PCNA, proliferating cell nuclear antigen; TIMP1, tissue inhibitor of metalloproteinases 1.

**Table 2:** Studies for CEA on the response to nCRT in rectal cancer.

Author (Reference)	Year	Number of Samples	Blood Collection	Cut off Values for CEA (ng/mL)	*p*-Value
Das et al. [92]	2007	562	pre-nCRT	≤2.5	*p* = 0.015
Yoon et al. [93]	2007	351	pre-nCRT	≤5	*p* = 0.004
Moreno Garcia et al. [94]	2009	148	pre-nCRT and post-surgery	≤2.5	*p* = 0.05
Kalady et al. [95]	2009	242	Not Reported	≤2.5	*p* = 0.19
Park et al. [96]	2009	352	pre and post-nCRT	≤3	*p* < 0.001
Lee et al. [97]	2009	490	pre-nCRT	≤5	*p* = 0.004
Kang et al. [98]	2010	84	pre and post-nCRT	≤3	*p* = 0.01
Aldulaymi et al. [99]	2010	33	pre-nCRT	≤5	*p* = 0.002
Yan et al. [100]	2011	98	pre-nCRT	≤3	*p* = 0.002
Hur et al. [101]	2011	37	pre-nCRT	≤3	*p* = 0.54
Moureau-Zabotto et al. [102]	2011	168	pre-nCRT	≤5	*p* = 0.019
Wallin et al. [103]	2013	267	pre-nCRT	≤3.4	*p* = 0.008
Restivo et al. [104]	2013	260	pre-nCRT	≤5	*p* = 0.001
Lee et al. [105]	2013	345	pre-nCRT	≤5	*p* = 0.002
Huh et al. [22]	2013	391	pre-nCRT	≤5	*p* = 0.002
Yeo et al. [106]	2013	609	pre-nCRT	≤5	*p* < 0.001
Yang et al. [107]	2013	138	pre-nCRT	≤6	*p* = 0.152
Wang et al. [108]	2014	240	pre-nCRT	≤5	*p* = 0.047
Zeng et al. [21]	2015	323	pre-nCRT	≤5	*p* = 0.007
Kim et al. [90]	2015	419	pre-nCRT	Not Reported	
Kleiman et al. [109]	2015	141	pre and post-nCRT	Not Reported	*p* = 0.003
Song et al. [91]	2016	1782	pre-nCRT	Not Reported	
Probst et al. [89]	2016	18,113	pre-nCRT	Not Reported	*p* < 0.001

Abbreviation: nCRT, neoadjuvant chemoradiation; CEA, carcinoembryonic antigen.

## Abbreviations

CRC, colorectal Cancer; nCRT, neoadjuvant chemoradiation; TRG, tumor regression grade; MSKCC, Memorial Sloan Kettering Cancer Center; AJCC, American Joint Committee on Cancer; RECIST, Response Evaluation Criteria in Solid Tumors; OS, overall survival; 5-FU, 5-Fluorouracil; DFS, disease free survival; MRI, Magnetic Resonance Imaging; TP53, tumor protein 53; KRAS, Kirsten rat sarcoma viral oncogene homolog; CIMP, CpG island methylator phenotype; ZNF160, zinc finger protein 160; XRCC2 and 3, X-ray repair cross-complementing protein 2 and 3; HFM1, ATP dependent DNA helicase homolog; ASXL2, additional sex combs-like protein 2; siRNA, small interfering RNA; AKR1C3, aldo-keto reductase family 1 member C3; CXCL9-11, C-X-C motif chemokine ligand 9-11; IDO1, indoleamine 2,3-dioxygenase 1; MMP-2, 9, and 12, matrix metalloproteinase-2, 9, and 12; HLA-DRA, HLA class II histocompatibility antigen, DR α chain; NPTX2, neuronal pentraxin-2; EGFR, epidermal growth factor receptor; VEGF, vascular endothelial growth factor; p21, protein 21; Bax, BCL2 associated X protein; Bcl2, B-cell CLL/lymphoma 2; COX-2, cyclooxygenase-2; HIF1-α, hypoxia-inducible factor 1-α; ATM, ataxia telangiectasia mutated; MRE11, meiotic recombination 11 homolog A; Paf15, PCNA-associated factor 15; Plk1, polo-like kinase 1; VRK1 and 2, vaccinia-related kinase 1 and 2; PCNA, proliferating cell nuclear antigen; GOLPH3, golgi phosphoprotein 3; FAK, focal adhesion kinase; FGFR4, fibroblast growth factor receptor 4; NF-κB, nuclear factor-κ B; APAF-1, apoptotic protease-activating factor 1; DSG3, desmoglein 3; TAGLN, transgelin; VNN1, vascular non-inflammatory molecule 1; TKT, transketolase; HADHA, hydroxyacyl-CoA dehydrogenase; HSD17B2, 17-β-hydroxysteroid dehydrogenase type 2; HMGCS2, 3-hydroxy-3-methylglutaryl coenzyme A synthase; TIMP1, tissue inhibitor of metalloproteinases 1; RAD51, DNA repair protein RAD51 homolog 1; HR MAS MRS, high-resolution magic angle spinning magnetic resonance spectroscopy; TNM, Tumor Node Metastasis; TILs, tumor-infiltrating lymphocytes; PD-L1, programmed cell death ligand 1; CTLA4, cytotoxic T-lymphocyte protein 4; CD4, -8, -40, -45, cluster of differentiation 4, 8, 40, 45; MDSCs, Myeloid-Derived Suppressor Cells; IHC, Immunohisto chemistry; CEA, carcinoembryonic antigen; CA19-9, carbohydrate antigen 19-9; CAIX, carbonic anhydrase 9; MALDI-TOF MS, Matrix-assisted laser desorption/ionization-time-of-flight mass spectrometer; HX, hypoxanthine; PEP, phosphoenolpyruvic acid; CTC, circulating tumor cells; CK20, cytokeratin-20; CCL-5, C-C motif chemokine ligand 5; DAPI, 4′,6-diamidino-2-phénylindole; cfDNA, cell-free DNA; IL-2, -4, -6, -8, -10, and -13, interleukin-2, -4, -6, -8, -10, and 13; IFN-γ, interferon-gamma; TNF-α, tumor necrosis factor-α; MCP-1, monocyte chemoattractant protein 1; TRAIL, TNF-related apoptosis-inducing ligand; NLR, neutrophil-to-lymphocyte ratio; d-NLR, derived neutrophil-to-lymphocyte ratio; PLR, platelet-to-lymphocyte ratio; NPS, neutrophil-platelet score; mGPS, the modified Glasgow prognostic score; SNP, single nucleotide polymorphism; TS, thymidylate synthase; EGF, epidermal growth factor; CCD1, coiled-coil protein DIX1; SOD2, superoxide dismutase 2; OGG1, 8-oxoguanine DNA glycosylase; MTHFR, methylenetetrahydrofolate reductase; SMAD3, SMAD family member 3; TRBP, trans-activation-responsive RNA-binding protein; DROSHA, double-stranded RNA-specific endoribonuclease; MUC17, mucin 17; SMUG1, single-strand-selective Monofunctional -DNA glycosylase 1.
